# Tracking fibrosis in myeloproliferative neoplasms by CCR2 expression on CD34^+^ cells

**DOI:** 10.3389/fonc.2022.980379

**Published:** 2022-08-22

**Authors:** Giulia Pozzi, Cecilia Carubbi, Giuliana Gobbi, Sara Tagliaferri, Prisco Mirandola, Marco Vitale, Elena Masselli

**Affiliations:** ^1^ Department of Medicine and Surgery (DiMeC), University of Parma, Parma, Italy; ^2^ Parma University Hospital, (AOU-PR), Parma, Italy

**Keywords:** myeloproliferative neoplasms, myelofibrosis, flow cytometry, CCR2, biomarkers, bone marrow fibrosis, diagnosis

## Abstract

In myeloproliferative neoplasm (MPNs), bone marrow fibrosis - mainly driven by the neoplastic megakaryocytic clone - dictates a more severe disease stage with dismal prognosis and higher risk of leukemic evolution. Therefore, accurate patient allocation into different disease categories and timely identification of fibrotic transformation are mandatory for adequate treatment planning. Diagnostic strategy still mainly relies on clinical/laboratory assessment and bone marrow histopathology, which, however, requires an invasive procedure and frequently poses challenges also to expert hemopathologists. Here we tested the diagnostic accuracy of the detection, by flow cytometry, of CCR2^+^CD34^+^ cells to discriminate among MPN subtypes with different degrees of bone marrow fibrosis. We found that the detection of CCR2 on MPN CD34^+^ cells has a very good diagnostic accuracy for the differential diagnosis between “true” ET and prePMF (AUC 0.892, *P*<0.0001), and a good diagnostic accuracy for the differential diagnosis between prePMF and overtPMF (AUC 0.817, *P*=0.0089). Remarkably, in MPN population, the percentage of CCR2-expressing cells parallels the degree of bone marrow fibrosis. In ET/PV patients with a clinical picture suggestive for transition into spent phase, we demonstrated that only patients with confirmed secondary MF showed significantly higher levels of CCR2^+^CD34^+^ cells. Overall, flow cytometric CCR2^+^CD34^+^ cell detection can be envisioned in support of conventional bone marrow histopathology in compelling clinical scenarios, with the great advantage of being extremely rapid. For patients in follow-up, its role can be conceived as an initial patient screening for subsequent bone marrow biopsy when disease evolution is suspected.

## Introduction

Classic Philadelphia-negative myeloproliferative neoplasms (MPN) include essential thrombocythemia (ET), polycythemia vera (PV) and myelofibrosis (MF). MF - arising *de novo* (primary MF, PMF) or evolving from an antecedent PV or ET (secondary MF, sMF) - is the most aggressive MPN, with the highest rate of leukemic transformation (1).

MPNs display a multifactorial pathogenesis due to both cell-intrinsic and extrinsic events. Cell-intrinsic factors include acquired somatic driver and non-driver mutations that provide a selective advantage to the neoplastic clone over normal hematopoietic stem cells and elicit, eventually, a myeloproliferative phenotype (2). In addition to somatic mutations, cell intrinsic events also list a number of germline genetic variants, recently emerging as determinants of the individual predisposition to develop an MPN, of the disease phenotype, response to therapy and outcome (3). MPN cells also exert cell extrinsic effects mainly attributable to the production of a plethora of pro-inflammatory cytokines, reactive oxygen species and growth factors which alter tissue homeostasis at both local (bone marrow niche) and systemic level (4, 5).

Key players in this process are neoplastic megakaryocytes (MKs), resulting from a process of aberrant MK proliferation and differentiation, particularly exacerbated in MF (6–8). MF MKs are hyperplastic and display typical morphological abnormalities such as tight clustering, cellular pleomorphism (presence of the so called “dwarf” MKs) and a peculiar transcriptional profile enriched in pro-inflammatory pathways as compared to normal MKs (9). Specifically, MF MKs are the primary cellular source of TGF-β1, which is the major driver of bone marrow (BM) fibrosis and collagen deposition (10, 11).

BM fibrosis is a central pathological feature of MPN contributing to a perturbed niche (the so called “bad soil”) that favors malignant over normal hematopoiesis (12). Indeed, it is well established that the degree of BM fibrosis correlates with adverse clinical features in PMF such as lower hemoglobin level, larger spleen, and higher percentage of peripheral blood blasts (13). Consistently, BM fibrosis grade >1 has been associated with shorter overall survival (median: 51 months) as compared to grade ≤1 (median: 147 months), indicating high grade of BM fibrosis as an independent prognostic factor to predict patient outcome (14). More in general, in MPNs, higher grade of fibrosis dictates a more severe disease stage with dismal prognosis and higher risk of leukemic evolution (15). Therefore, accurate patient allocation into different disease categories and timely identification of fibrotic transformation are mandatory for adequate treatment planning, including bone marrow transplant for eligible patients. Diagnostic strategy still mainly relies on clinical assessment and BM histopathology, which, however, requires an invasive procedure and frequently poses challenges also to expert hemopathologists (16).

During the past two decades, different grading systems have been proposed to evaluate BM fibrosis in pathological conditions, most of them deriving from the Bauermeister scale (17). In 2005, a panel of expert pathologists published the European Consensus on grading of BM fibrosis, in which they outlined the guidelines for bone marrow histological analysis and distinguished four categories based on the qualitative (reticulin or collagen) and quantitative evaluation of fibrotic tissue (18). However, these grading systems are semi-quantitative, and suffer major limitations related to subjectivity (19). Histological evaluation is further hampered by the heterogeneity of fibrotic area within a single sample, the variability of pre-analytical and staining process, subjective assessment due to the lack of an internal standard of positive staining (19–21). Therefore, novel strategies aimed to improve MPN diagnostic algorithm are eagerly awaited.

We recently demonstrated that the CCL2/CCR2 chemokine system plays an important role in PMF pathophysiology. Indeed, higher levels of CCL2 in PMF, sustained by homozygosity for the G/G rs1024611 genotype, boost cell-intrinsic pro-survival signals *via* Akt phosphorylation due to the selective overexpression of CCR2 by PMF hematopoietic progenitors (22, 23). Moving from these findings, here we investigated the diagnostic accuracy of flow-cytometric detection CCR2-positive CD34^+^ cells in tracking fibrosis in MPNs.

## Methods

### Study population

The study was approved by the local ethical committee (Comitato Etico Area Vasta Emilia-Romagna, Prot. 11537-14/03/2019). Overall, 66 MPN patients were enrolled at the Hematology and BMT Unit of Parma University Hospital, of which 24 ET, 17 prePMF, 9 overtPMF, 6 sMF and 4 unclassifiable MPN (MPN-u). Diagnosis was posed according to the WHO 2016 and IWG-MRT criteria (24, 25). 3 PV and 4 additional ET patients underwent disease re-assessment for suspected evolution into sMF but did not fulfill the IWG-MTR for post-PV/ET MF. These patients were indicated as ET and PV with fibrosis grading 0-1 (ET/PV-F). One of these patients eventually developed after 3 years, a post-PV MF. Therefore, it was listed both in the ET/PV-F and sMF groups according to the disease stage. The only cytoreductive treatment allowed was hydroxyurea. Demographical, clinical, and biological characteristics at the time of sampling were collected from patients’ clinical records and summarized in **Table 1**.

**Table 1:** Demographic, clinical, biological and histopathological characteristic of MPN patients included in the study.

	ET(N. 24)	prePMF(N. 17)	overtPMF(N. 9)	sMF(N. 6)	ET/PV-F (N. 7)	MPN-u (N. 4)	*P* value
Age (sampling)							
mean (range), yrs	59 (19-83)	64 (34-83)	69 (37-84)	73 (57-90)	57 (45-79)	63 (41-74)	n.s.
Follow-up							
mean (range), yrs	1 (0-26)	3 (0-21)	4 (1-10)	11 (9-22)	10 (7-23)	1 (0-1)	n/p
Male							
N. (%)	7 (29.2)	8 (47.1)	5 (55.5)	3 (50.0)	5 (71.4)	1 (25.0)	n.s.
Hb							
median (range), g/dL	13 (10.8-15.0)	12.5 (7.6-215.2)	11 (7.1-13.3)	12.7 (10.5-13.9)	12.9 (10.2-16.7)	12.7 (9.6-13.3)	P < 0.05 ET vs. overtPMF
WBC							
median (range), x10^9^/L	7.4 (5.2-10.4)	7.2 (4.1-13.3)	6.8 (3.7-20.2)	12.3 (2.5-34.2)	10.0 (3.7-13)	8.7 (6.2-19.8)	n.s.
PLT							
median (range), x10^9^/L	684 (416-1386)	623 (97-1526)	444 (380-663)	311 (80-1915)	616 (487-921)	333.5 (179-683)	*P* < 0.05 ET vs. overtPMF
Spleen Ø							
mean (range), cm	10 (9-15)	15 (10-18)	14 (9-19)	11.5 (8.2-17.5)	15 (11-20)	14.5 (8.6-16.5)	*P* < 0.01 ET *vs.* prePMF
LDH							*P* < 0.05 ET vs. prePMF *P* < 0.05 ET *vs.* overtPMF *P* < 0.05 ET vs. sMF *P* < 0.01 MPN-u *vs.* prePMF *P* < 0.01 MPN-u vs. overtPMF *P* < 0.01 MPN-u *vs.* sMF
median (range), mU/mL, x ULN	1.0 (0.8-5.1)	2.1 (0.9-5.1)	2.3 (1.4-6.3)	1.9 (1.3-5.2)	2.1 (1.9-2.2)	0.8 (0.7-1.3)	
PB Blast %							
median (range)	0 (0-0)	0 (0-4)	1 (0-6)	0 (0-15)	0 (0-4)	0 (0-2)	n.s.
Constitutional symptoms							
N. (%)	3 (12.5)	4 (23.5)	3 (33.3)	2 (33.3)	3 (42.8)	1 (25)	n.s.
Thrombosis/hemorrhage							
N. (%)	4 (16.7)	2 (11.8)	3 (33.3)	2 (33.3)	1 (14.3)	1 (25)	n.s.
IPSET							
Low N. (%) Int N. (%) High N. (%)	10 (41.6) 4 (16.8) 10 (41.6)	n/a	n/a	n/a	n/a	n/a	n/p
IPSS/DIPSS							
Low N. (%) Int-1 N. (%) Int-2 N. (%) High N. (%)	n/a	7 (41.2) 7 (41.2) 3 (17.6) 0 (0.0)	2 (22.2) 3 (33.4) 2 (22.2) 2 (22.2)	n/a	n/a	n/a	n.s. pre vs. overtPMF
MY-SEC							
Low N. (%) Int-1 N. (%) Int-2 N. (%) High N. (%)	n/a	n/a	n/a	1 (20.0) 3 (60.0) 0 (0.0) 1 (20.0)	n/a	n/a	n/p
BM fibrosis grading							
MF-0 N. (%) MF-0-1 N. (%) MF-1 N. (%) MF-2/3 N. (%)	n/a	1 (5.9) 4 (23.5) 12 (70.6) 0 (0.0)	0 (0.0) 0 (0.0) 0 (0.0) 9 (100.0)	0 (0.0) 0 (0.0) 0 (0.0) 6 (100.0)	1 (14.3) 2 (28.6) 4 (57.1) 0 (0.0)	2 (50.0) 1 (25.0) 1 (25.0) 0 (0.0)	*P* < 0.0001 pre vs. overtPMF and vs. sMF *P* < 0.0001 ET/PV-F vs overtPMF *P* = 0.001 ET/PV-F vs sMF *P* = 0.001 MPN-u vs overtPMF *P = *0.004 MPN-u vs sMF
Driver Mutations							
Tested pts *JAK2*V617F N. (%) *CALR* N. (%) *MPL* N. (%)	all 12 (50.0) 3 (12.5) 1 (4.2)	all 7 (41.2) 8 (47.1) 0 (0.0)	all 5 (55.6) 2 (22.2) 0 (0.0)	N. 5 4 (80.0) 0 (0.0) 1 (20.0)	all 4 (57.1) 3 (42.9) 0 (0.0)	all 3 (75.0) 1 (25.0) 0 (0.0)	*P* = 0.03 *CALR* ^mut^ ET vs. prePMF
Non-Driver Mutations							
Tested pts *TET2*, N. *DMNT3A*, N. *ASXL1*, N. *IDH1/2*, N. *EZH2*, N. *SRSF2*, N. *PTPN11*, N. *CSFR3R*, N. *NRAS/KRAS*, N. *U2AF1*, N. *TP53*, N. *RUNX*, N. *CEBPA*, N. *ETV6*, N.	N. 2 0 0 0 0 0 0 0 0 0 0 0 0 0 0	N. 5 3 0 3 0 1 0 0 1 0 0 0 0 0 0	N. 2 1 0 1 0 0 1 0 0 0 0 0 0 0 1	N. 4 2 1 0 0 0 0 1 0 1 0 1 0 0 0	N. 5 0 0 2 0 0 0 1 1 1 1 1 1 1 0	N. 2 0 0 1 0 1 0 0 0 0 0 0 0 0 0	n/p
Outcome							
Alive Deceased HSCT	24 (100.0) 0 (0.0) 0 (0.0)	13 (92.6) 3 (17.6)* 2 (11.7)	5 (55.6) 2 (22.2) 2 (22.2)	4 (66.7) 2 (33.3) 0 (0.0)	5 (71.4) 2 (28.6) 0 (0.0)	4 (100.0) 0 (0.0) 0 (0.0)	n.s.

^*^Including one death after HSCT.

BM, bone marrow; Hb, hemoglobin, HSCT, hematopoietic stem cell transplant; LDH, lactate dehydrogenase; IPSET, International Prognostic Score of Thrombosis for ET; IPSS/DIPSS, International Prognostic Scoring System/Dynamic International Prognostic Scoring System; MY-SEC, Myelofibrosis Secondary to PV and ET-Prognostic Score; n/a, not applicable; n/p, statistical analysis not performed; n.s. non-statistically significant; PB, peripheral blood; PLT, platelet count; pts, patients; WBC, white blood cell count. (Statistical analysis was performed by χ2/Fisher exact test and Kruskal-Wallis followed by Dunn’s test, as applicable).

### Hematopoietic stem cell isolation and flow cytometric analysis

15-20mL of peripheral blood/BM aspirate were collected in EDTA tubes. After mononuclear cells separation by Ficol-Hypaque stratification, primary hematopoietic stem cells, identified as CD34^+^ cells, were isolated by immunomagnetic positive selection (CD34 MicroBead Kit, Miltenyi Biotec, Bergisch Gladbach, Germany) and subsequently tested for CCR2 expression as previously described (23). Flow-cytometric (FCM) data analysis was performed by using the Kaluza Analysis Flow Cytometry Software (Beckman Coulter, Brea, CA, USA). Gating strategy was as follows: cell population was identified based on its SSC/FSC properties [FSC-(A)/SSC-(A) dot plot]; then CD34^+^ cells were gated and CD34^+^ cells with an equal area and height were selected to accurately remove clumps [greater FSC(A) relative to FSC(H)] and debris (very low FSC). Then, a standard gate for CD34^+^CCR2^+^ cells was created and the percentage of CD34^+^CCR2^+^ cells was normalized to the total percentage of CD34^+^ cell population for each sample (% CCR2^+^CD34^+^/CD34^+^, hereinafter indicated as CCR2^+^ cells).

### Statistical analysis

For statistical analysis, differences in the categorical variables were analyzed by χ2/Fisher exact test, while Mann-Whitney, or Kruskal Wallis test followed by Dunns’s test were performed for continuous variables, as applicable. Receiver-operating characteristic (ROC) curves of flow cytometry evaluation of CCR2 expression on primary CD34^+^ cells were generated to assess the diagnostic accuracy in terms of specificity and sensitivity to discriminate prePMF patients from trueET and overtPMF from prePMF. The area under the ROC curve (AUC) values were evaluated as recommended by Hanley et al. (26). Correlation between the percentage of CCR2-expressing CD34^+^ cells and circulating blasts, grading of bone marrow fibrosis and prognostic score were performed by Spearman correlation test. All statistical analyses were performed using Prism 9 (GraphPad software San Diego, CA, USA), and only differences with a *P* value < 0.05 were considered statistically significant.

## Results

### Diagnostic performance of CCR2^+^ cell detection by FCM in discriminating trueET vs. prePMF and prePMF vs. overtPMF

We previously demonstrated that CD34^+^ cells from PMF patients uniquely expressed CCR2 which, by contrast, is nearly absent on CD34^+^ cells from healthy subjects and other MPN subtypes (23). Moving from these findings we tested whether FCM detection of CCR2-expressing CD34^+^ cells could represent an accurate diagnostic tool for the differential diagnosis of MPN subtypes with different degrees of bone marrow fibrosis, with a focus on compelling clinical scenarios, i.e. prePMF vs “true” ET and overtPMF vs prePMF. For this purpose, freshly isolated CD34^+^ cells from ET (N. 24), prePMF (N.17) and overtPMF (N.9) were tested for CCR2 expression by FCM.

**Figure 1:**
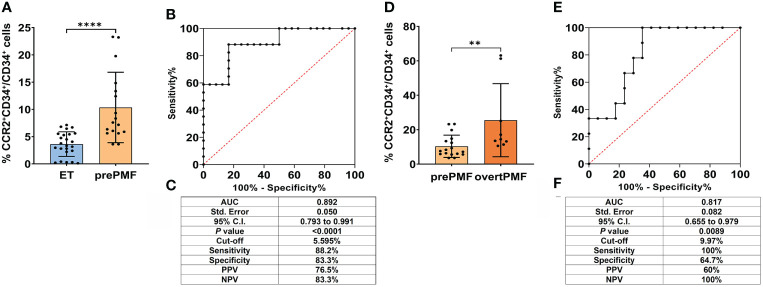
Diagnostic accuracy of flow cytometry evaluation of CCR2 expression on CD34+ cells in ET *vs.* prePMF and prePMF *vs.* overtPMF. **(A)** CCR2 expression by FCM on CD34^+^ cells isolated from ET (N = 24) and prePMF patients (N = 17). CCR2 expression is reported as % of CCR2^+^CD34^+^/CD34^+^ cells and data are showed mean ± SD (****, *P* < 0.001, Mann-Whitney test). **(B)** ROC curve of FCM analysis of CCR2-expressing CD34+ cells in ET and prePMF patients. **(C)** Summary table reporting: Area under the curve (AUC), 95% confidential interval (95% CI), *P* value (P), cut-off value, sensitivity, specificity, positive predictive value (PPV) and negative predictive value (NPV). **(D)** CCR2 expression by FCM on CD34^+^ cells isolated from prePMF (N = 17) and overtPMF patients (N = 9). CCR2 expression is reported as % of CCR2^+^CD34^+^/CD34^+^ cells and data are showed mean ± SD Mann-Whitney test). **(E)** ROC curve of FCM analysis of CCR2-expressing CD34+ cells in prePMF and overtPMF patients. **(F)** Summary table reporting: Area under the curve (AUC), 95% confidential interval (95% CI), *P* value (P), cut-off value, sensitivity, specificity, positive predictive value (PPV) and negative predictive value (NPV).

Consistently with our previous findings, the percentage of CCR2^+^ cells was significantly higher in prePMF as compared to ET (% of CCR2^+^CD34^+^/CD34^+^ cells: 3.62 ± 2.26 in trueET *vs.* 10.35 ± 6.46 in prePMF, *P* < 0.0001, **Figure 1A**). The analysis of CCR2 expressing CD34^+^ cells generated a ROC curve with an area under the curve (AUC) of 0.892 (95% CI: 0.793–0.991, P<0.0001 (**Figure 1B**). The optimal cut-off value was 5.595% with a sensitivity of 88.24% and a specificity of 83.3%, a positive predictive value (PPV) of 76.5% and a negative predictive value (NPV) of 83.3% (**Figure 1C**). Overall, these data indicate that flow cytometry detection of CCR2^+^ cells has a very good diagnostic performance in discriminating patients with prePMF vs trueET, which often share a similar clinical phenotype.

We then asked whether the flow cytometry evaluation of CCR2^+^ cells could be effective also in discriminating the two PMF subtypes: prePMF vs overtPMF. Consistently with our published data (23), overtPMF CD34^+^ cells display a significantly higher CCR2 expression as compared to prePMF (% of CCR2^+^CD34^+^/CD34^+^ cells: 10.35 ± 6.46 in prePMF *vs.* 25.53 ± 21.23 in overtPMF, *P* =0.0076, **Figure 1D**). With the limits of a smaller sample size, ROC curve showed an AUC of 0.817 (95% CI: 0.655–0.979, *P*=0.0089) (**Figure 1E**). The optimal cut-off value of 9.970% is associated to a specificity of 64.7% and a sensitivity of 100%, with a PPV of 60% and a NPV of 100% (**Figure 1F**). Therefore, flow cytometry detection of CCR2^+^ cells revealed a good diagnostic performance for discriminating patients with prePMF vs overtPMF. Of note, the two patient cohorts shared a similar phenotype in terms of CBC count, circulating blast percentage, LDH levels, spleen size, presence of constitutional symptoms, history of thrombosis/hemorrhage and IPSS/DIPSS risk category (see **Table 1**), implying that CCR2^+^ cell percentage could be extremely informative in clinical settings with overlapping disease presentations.

OvertPMF cohort clearly includes two outliers with an elevated percentage of CCR2^+^ cells (% CCR2^+^CD34^+^/CD34^+^ = 63.1 and 61.3%). Of note, also upon the exclusion of these samples, the ROC curve shows a statistically significant AUC (0.765, P<0.05), maintaining a fair diagnostic accuracy. Intriguingly, these two patients harboring extremely elevated levels of CCR2^+^ cells, were either enriched in HMR mutations or carrying an unfavorable (complex) karyotype. Both patients displayed an increased percentage of circulating blasts and a high DIPPS score.

### CCR2^+^ cell percentage is capable to track disease fibrotic changes and correlates with the degree of bone marrow fibrosis in MPNs

**Figure 2:**
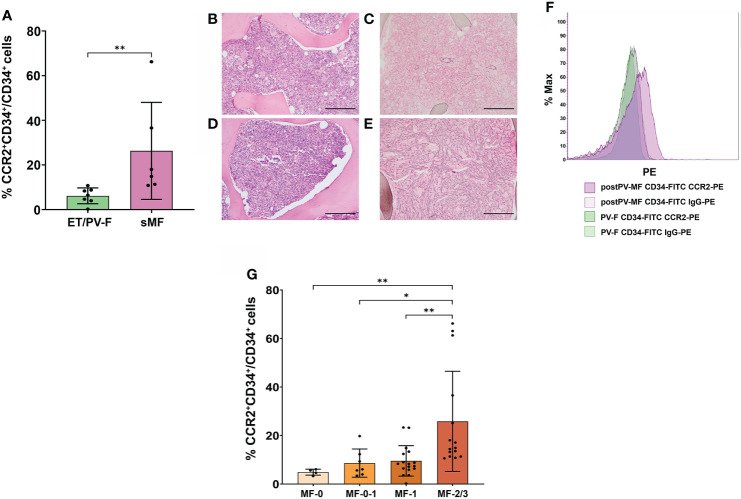
Correlation between CCR2 expression on CD34^+^ cells and bone marrow fibrosis in MPNs. **(A)** Percentage of CCR2-expressing CD34^+^ cells, identified as CCR2^+^CD34^+^/CD34^+^ cells by FCM, in ET/PV-F (N.7) and sMF (N.6) (**, *P *= 0.0012, Mann-Whitney Test). **(B‐E)** BM histopathology during PV-F and post-PV MF phases. Hematoxylin-Eosin staining **(B, D)** and reticulin staining **(C, D)** Magnification 10x. Scale bar=1mm. **(F)** FCM histograms showing the percentage of CCR2-expressing CD34^+^ cells during PV with mild BM fibrosis (PV-F) phase (in green) and during post-PV MF phase (in violet). **(G)** Percentage of CCR2-expressing CD34^+^ cells, identified as CCR2^+^CD34^+^/CD34^+^ cells by FCM, in MPN patients stratified according to the grading of bone marrow fibrosis in fibrosis in MF-0 (N. 4), MF-0-I (N. 7), MF-I (N. 17) and MF-2/3 (N.15) (*, *P* < 0.05; **, *P* < 0.01 by Kruskal Wallis followed by Dunn’s Test).

We then asked whether FCM detection of CCR2^+^ cells could be used as a diagnostic tool to track the progression of bone marrow fibrosis in MPNs. CCR2 expression was evaluated in 13 ET/PV patients who underwent BM reassessment for suspected evolution into a spent phase (sMF) because of increase in spleen size and/or onset of constitutional symptoms and/or increase in LDH levels. Diagnosis of sMF was confirmed by BM histopathology (fibrosis grading ≥ 2) in 6 out of 13 patients, whilst for 7 patients only a mild progression of fibrosis (fibrosis grading 0 or 1) was detected, thus not fulfilling IWG-MRT criteria for classification into post-ET/PV sMF (25). We found that the percentage of CCR2^+^ cells was significantly higher in patients who received a diagnosis of sMF as compared to those who reveled only slight fibrosis progression and therefore, could not be allocated into post-PV/ET disease category (ET/PV-F) (*P*=0.0012, **Figure 2A**). We further proved the clinical relevance of CCR2^+^ cells detection by FCM during the longitudinal follow-up of a PV patient who underwent a first bone marrow re-assessment in 2019, when transition into post-PV was suspected upon increased spleen size (longitudinal diameter of 14.5 cm vs 12.8 cm at PV diagnosis), increased LDH levels (1.4 UNL) and worsening leukocytosis. At that time, BM histopathology showed trilinear hyperplasia with loose clusters of large, hyperlobulated megakaryocytes (**Figure 2B**) and reticulin staining revealed mild (MF-1) fibrosis (**Figure 2C**). Diagnosis of PV with fibrosis grade MF-1 was posed. At this point, CCR2 FCM expression on BM CD34^+^ cells tested negative (only 0.22% of cells expressed CCR2 (**Figure 2F**). Two years later, the patient was re-evaluated because of further increase in white blood cell count, spleen size (longitudinal diameter of 16.4 cm), paralleled by a marked decrease in platelet count and hemoglobin. At this point, BM histopathology revealed a further increase in BM cellularity with trilinear hyperplasia (**Figure 2D**) and fibrosis grade MF-2, as documented by the reticulin staining (**Figure 2E**), now eventually matching the diagnostic criteria for post-PV MF. Interestingly, CCR2-expressing CD34^+^ cells showed a > 50-fold increase (11.8% CCR2^+^CD34^+^ cells) as compared to the previous sampling (**Figure 2F**).

These data indicate that the % of CCR2^+^ cells in bone marrow samples could be informative on the probability of disease progression.

On these bases, we investigated whether CCR2^+^ cell percentage could be correlated to the degree of bone marrow fibrosis in MPNs. For this purpose, we analyzed all MPN patients with fibrosis (i.e., pre- and overt-PMF, sMF, ET/PV-F and MPN-u); ET were excluded because of lack of reticulin fibers at the onset [as per by WHO criteria (1)]. According to the European Consensus on grading of bone marrow fibrosis (26), MPN patients were stratified in four categories including MF-0, MF-1, MF-2 and MF-3. As some patients were annotated with fibrosis grading 0-1, we considered them as a separate group in the analysis, while the only patient matching MF-3 criteria was grouped with MF-2 patients. We found that the percentage of CCR2^+^ cells significantly increased with the grading of bone marrow fibrosis (r = 0.656, P< 0.0001, Spearman correlation). Indeed, MF-2/3 patients displayed a significantly higher percentage of CCR2-expressing CD34^+^ cells as compared to the other subgroups (*P*=0.0017 *vs.* MF-0, *P*= 0.024 vs. MF-0-1 and *P*=0.0089 *vs.* MF-1 respectively) (**Figure 2G**). Overall, these data pinpoint CCR2^+^ cells as a novel biomarker of BM fibrosis.

### CCR2 expression on CD34^+^ cells is associated to higher risk categories and presence of circulating blasts in MF

**Figure 3:**
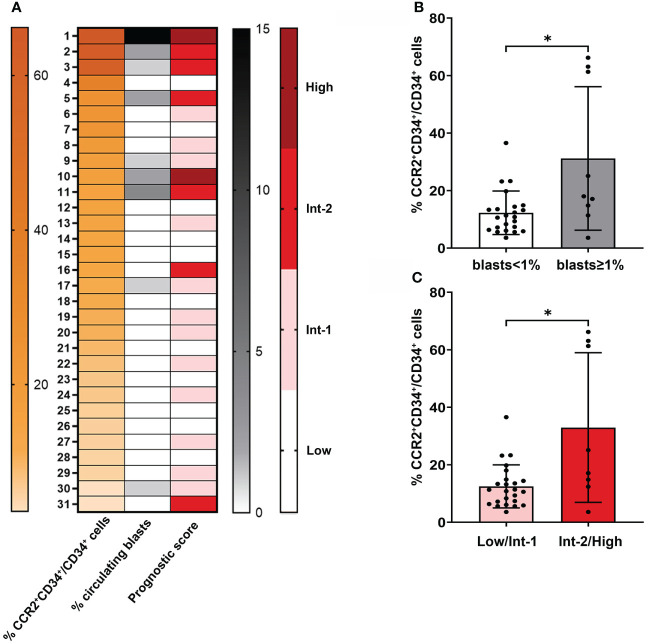
Correlation between CCR2 expression on CD34+ cells and the presence of circulating blasts and prognostic score in MF. **(A)** Heatmap generated from flow cytometric and clinical data of 31 MF patients. The first column (orange scale) represents decreasing CCR2^+^CD34^+^/CD34^+^ cell percentage values, the second and third column the percentage of circulating blasts (gray scale) and prognostic risk score (red scale) for each corresponding patient. **(B)** Percentage of CCR2-expressing CD34^+^ cells in MF patients with circulating blast <1% and ≥1%. Data are shown as mean ± SD (*P = 0.015, Mann-Whitney Test). **(C)** Percentage of CCR2-expressing CD34^+^ cells in MF patients in the low/Int-1 and Int-2/High prognostic risk category. Data are shown as mean ± SD (*P = 0.026, Mann-Whitney Test).

Among MPNs, MF (including both PMF and sMF) is typified by a more aggressive clinical course and worse prognosis (1). Here we wanted to assess whether CCR2 expression on CD34^+^ cells could be associated to adverse clinical features in the MF population, such as the presence of circulating blasts and the composite risk scores IPSS/DIPSS for PMF and MYSEC-PM for sMF. With the exception of one sMF patient that was excluded from the analysis because of the absence of driver-mutation status, all MF patients (prePMF, overtPMF and sMF, N=31) were evaluated. As shown in **Figure 3A**, MF patients displaying the highest levels of CCR2^+^ cells were enriched in circulating blasts and were mainly allocated into the higher risk categories, demonstrating a significant correlation with circulating blasts and prognostic scores (r=0.481, *P*=0.006; r=0.472, *P*=0.0074 respectively, Spearman correlation). Indeed, the percentage of CCR2-expressing CD34^+^ cells was significantly increased in patients with (≥ 1%) *vs.* without (<1%) circulating blasts (*P*=0.015, **Figure 3B**) and in intermediate-2/high *vs.* low/intermediate-1 risk patients (*P*=0.026, **Figure 3C**), suggesting that CCR2 expression on CD34^+^ cells is a predictive factor of poor prognosis.

## Discussion

Differential diagnosis among MPN entities has significant prognostic implications, but still poses several challenges due to overlapping clinical, laboratory and molecular features (27). BM histopathology, although of critical importance, is undermined by many drawbacks as the lack of pathognomonic morphological features, suboptimal performance in specific clinical setting and the invasive procedure (19, 21).

One of the main diagnostic challenges is represented by the differential diagnosis between true ET and prePMF. Lack of fibrosis in the early phases and onset with isolated thrombocytosis can lead to prePMF being misdiagnosed as ET (28). Indeed, median age at diagnosis is similar (56 and 57 years, respectively), and both populations are more often female. Although the rate of palpable splenomegaly seemed significantly higher in prePMF cases (23% *vs.* 16%) this could not be used to discriminate the two. Despite subtle differences in leukocytes, platelets, hemoglobin and LDH level may be of help, clinical and laboratory features are, at best, only able to differentiate 40–50% of patients (29). Nevertheless, an accurate patient allocation is critical since prePMF displayed a reduced overall survival as compared to ET (30). Additionally, several studies highlighted the higher rate of hemorrhagic complications in prePMF (31, 32).

We demonstrated that FCM detection of CCR2^+^ cells had a very good diagnostic performance in discriminating patients with prePMF vs true ET (AUC=0.892), with a cut-off value of 5.595% CCR2+ cells. Of note, consistently with the literature, ET and prePMF of our cohort had similar demographic characteristics while differed in spleen size (median longitudinal diameter in ET 10 *vs.* 15 cm in prePMF, *P*<0.01 Kruskal Wallis followed by Dunn’s test), LDH levels (median LDH in ET 1.0 x ULN vs. 2.1 x ULN in prePMF, *P<*0.05 by Krukal Wallis followed by Dunn’s test) and frequency of *CALR* mutations (12.5% in ET vs 47.1% in prePMF, *P*=0.03 by Fisher’s exact test) (see **Table 1**), being therefore representative of larger patient cohorts.

FCM detection of CCR2^+^ cells proved also effective - although with a lower performance (AUC=0.817, cut-off value 9.97%) - in discriminating among pre vs overtPMF. Of note, the NPV of 100% indicate that when the percentage of CCR2^+^ cells is below 9.97%, the probability of dealing with an overt disease is 0%, providing thus a sensitivity of 100%.

OvertPMF differs from prePMF in several clinical and biological aspects, including more pronounced disease manifestations, adverse mutation profile, and worse outcome. The main WHO parameter that distinguishes these two disease entities relies in the higher grading of BM fibrosis that typifies overtPMF (MF≥2). However, grading assessment could be hampered by the heterogeneity of fibrotic areas within a single sample. In contrast with the literature, prePMF and overtPMF of our cohort had a similar phenotype (probably due to the limited sample size of overtPMF cohort and the limited number of patients tested for HMR). However, the fact that FCM CCR2 testing is capable to discriminate with a good diagnostic accuracy the two disease categories despite the overlapping clinical presentation, further support the potential utility of this assay in not univocal clinical scenarios.

We also demonstrated that the percentage of CCR2^+^ cells is significantly different in ET and PV patients with mild fibrosis (ET/PV-F) as compared to those evolved into spent phase (MF≥2). With the limitations due to the sample size, we can conclude that, despite a similar clinical picture suggestive of disease evolution, only those patients fulfilling the criteria of postPV/ET MF displayed significantly increased levels of CCR2^+^ cells. This prompted us to ask whether the percentage of CCR2^+^ cells correlated with the degree of BM fibrosis (MF 0, 0-1, 1 and 2/3) in a broader MPN patient cohort that included compelling disease entities such as, as a matter of fact, ET/PV-F and MPN-u, with or without fibrosis, in addition to prePMF, overtPMF and sMF, for a total of 40 MPN patients. Indeed, we found that the percentage of CCR2^+^ cells significantly increases with the grading of bone marrow fibrosis (r=0.656 P< 0.0001, Spearman correlation). Finally, the observation that, during the longitudinal follow-up of a MPN patient, the increase in CCR2^+^ cells paralleled disease progression into post-PV MF provides the proof-of-concept of the potential impact in the clinical setting of our findings.

It is well known that, in MPN, higher grade of fibrosis determines a more severe disease stage with higher risk of leukemic evolution (13, 32). Consistently, we found that in MF (prePMF + overtPMF + sMF), those patients expressing higher percentages of CCR2^+^ cells were enriched in PB blasts and allocated in the higher risk categories. Similarly, the two outliers in the overtPMF group display unfavorable genetic features such as either presence of HMR mutations or complex karyotype. Thus, we can envision that the expression of CCR2 by the MPN hematopoietic stem cell progenitors coincides with the acquisition of aggressive biological features. Of course, larger cohorts of molecularly annotated patients are required to confirm this preliminary observation.

MPNs are a group of hematologic malignancies characterized by phenotypic mimicry and transformation to each other (33). Thus, novel strategies aimed to improve the diagnostic accuracy in MPN are mandatory in order to optimize personalized treatment plannings.

With all the limitations due to the small sample size, this study identifies a novel diagnostic biomarker in MPNs, and opens the avenue for further studies in larger cohorts of patients to validate FCM detection of CCR2+ positive cells as tool to track fibrotic changes.

FCM detains relevant advantages as compared to other diagnostic tools, first of all the rapid and complete characterization in a single-shot analysis. Thus, notwithstanding the pivotal and indispensable role of BM morphological evaluation by histopathology, FCM CCR2 detection may offer a fast clinical orientation. Moreover, FCM can be performed also on peripheral blood samples: this implies that, in case of suspected disease evolution into spent phase, peripheral blood CCR2^+^ cell determination may be offered as an initial non-invasive screening for subsequent identification of those patients who deserve further assessment by marrow biopsy.

## Data availability statement

The raw data supporting the conclusions of this article will be made available by the authors, without undue reservation.

## Ethics statement

The studies involving human participants were reviewed and approved by Comitato Etico Area Vasta Emilia-Romagna, Prot. 11537-14/03/2019. The patients/participants provided their written informed consent to participate in this study.

## Author contributions

EM, MV, and CC conceived the study. GP and CC performed experiments. GP, CC, and EM analysed data. EM and GP: manuscript writing. GG, PM, and MV: manuscript review and editing. ST advised statistics. All authors contributed to the article and approved the submitted version.

## Funding

Fondi Locali per la Ricerca to EM (MASSELLI_E_FIL).

## Acknowledgments

We are grateful to Cristina Micheloni, Luciana Cerasuolo and Vincenzo Alberto Piero Palermo for their technical support.

## Conflict of interest

The authors declare that the research was conducted in the absence of any commercial or financial relationships that could be construed as a potential conflict of interest.

## Publisher’s note

All claims expressed in this article are solely those of the authors and do not necessarily represent those of their affiliated organizations, or those of the publisher, the editors and the reviewers. Any product that may be evaluated in this article, or claim that may be made by its manufacturer, is not guaranteed or endorsed by the publisher.
